# Subphrenic Abscess

**Published:** 1898-03-19

**Authors:** 


### SUBPHRENIC ABSCESS.

A subphrenic abscess may be defined as a localised collection of pus, or pus and gas, between the inferior aspect of the diaphragm and the adjacent surface of the liver. It is a condition the occurrence of which is of extreme importance, from the difficulties which sometimes stand in the way of its diagnosis, especially in its early stages; from its liability to be mistaken for other affections; from its danger to life; and from the comparatively grave nature of the operative measures which have to be undertaken for its relief. Dealing with this disease in the Erasmus Wilson lectures, Mr. Waring first points out that the facts that the liver is only partially covered with peritoneum, and that the uncovered portion lies for the most part in direct contact with the lower surface of the diaphragm, have an important influence on the development of subphrenic abscess, for this may be of the nature of a localised peritonitis affecting the diaphragmatic surface of the liver where it is covered with peritoneum, or it may be an abscess in the connective tissue in connection with the uncovered portion.

In the intraperitoneal variety, which is more common than the extraperitoneal, the inferior boundary of a subphrenic abscess is usually formed of the superior surface of the right lobe of the liver, although sometimes both lobes may be involved. When both lobes enter into the formation of the abscess, this is usually extraperitoneal, although in some cases it would seem that an intraperitoneal abscess has spread through the falciform ligament. As to the origin of the disease, it may arise from injury of the hepatic region, but most commonly it arises from ulceration or septic inflammation of one or other of the hollow viscera. Ulcer of the stomach, of the duodenum, of the cæcum and its appendix, and, rarely, of the small intestine, inflammatory affections of the kidney, of the gall-bladder and ducts, suppuration of hydatid cysts, and collections of pus in the pleura, may all give rise to abscess under the diaphragm. The most common cause is ulceration of the stomach.

In regard to diagnosis, the disease is likely to be mistaken for an abscess in the upper portion of the liver, a solid tumour of the liver, a subphrenic hydatid cyst, a pleural effusion, and, when it contains gas, for a pyo-pneumothorax. In subphrenic abscess there is often a history of abdominal symptoms and absence of expectoration, whilst in pleural effusion the reverse is generally the case. Again, the heart is not generally displaced to any extent in subphrenic abscess, while in pleural effusion or pyo-pneumothorax this displacement is usually a marked physical sign. The auscultatory signs also are different. In subphrenic abscess the vesicular breath-sounds can be heard a variable distance down from the clavicle, usually as far as the third space, and below this level there is amphoric sound when the abscess contains gas, and dulness when it contains pus alone; while in pneumothorax the vesicular breath-sounds are absent on the affected side.

It has been said by some that when, on exploring with an aspirator, pus is found and the needle moves up and down with the respiration, a subphrenic abscess is present—the movement being due to the needle being fixed in the diaphragm. This, however, must not be taken as a pathognomonic sign, for the needle may be stuck in the thick walls of an abscess, and not in the diaphragm at all; while on the other hand, if a subdiaphragmatic abscess be sufficiently large to push the diaphragm upwards to any considerable extent the needle may pass into the cavity, and have no movements communicated to it. It may, in fact, be quite impossible to make a diagnosis between an encysted purulent collection of the basal portion of the chest and a subphrenic abscess.

The treatment of the disease is surgical. The abscess may be reached by three different routes—through the overlying portion of the abdominal wall, immediately below the costal margin; or through the overlying portion of the thoracic wall by resections of portions of one or more ribs, and incision of the pleura and diaphragm; or from the lumbar region, by an incision which is directly below the lower border of the last rib. The locality and connection of the abscess must determine which of these routes be adopted, but where it can be arranged the incision through the abdominal wall seems the best, and the most likely to be followed by a successful result.

As to results, the disease is a grave one in any case. According to Lang, who has collected 176 cases from surgical literature, the percentage of recoveries after operation is 47·9 per cent., and of those not operated on 12·3 per cent.

